# Structural characterization of eRF1 mutants indicate a complex mechanism of stop codon recognition

**DOI:** 10.1038/srep18644

**Published:** 2016-01-04

**Authors:** Shubhadra Pillay, Yan Li, Leo E Wong, Konstantin Pervushin

**Affiliations:** 1 School of Biological Sciences, Nanyang Technological University, 60 Nanyang Drive, 637551, Singapore

## Abstract

Eukarya translation termination requires the stop codon recognizing protein eRF1. In contrast to the multiple proteins required for translation termination in Bacteria, eRF1 retains the ability to recognize all three of the stop codons. The details of the mechanism that eRF1 uses to recognize stop codons has remained elusive. This study describes the structural effects of mutations in the eRF1 N-domain that have previously been shown to alter stop codon recognition specificity. Here, we propose a model of eRF1 binding to the pre-translation termination ribosomal complex that is based in part on our solution NMR structures of the wild-type and mutant eRF1 N-domains. Since structural perturbations induced by these mutations were spread throughout the protein structure, residual dipolar coupling (RDC) data were recorded to establish the long-range effects of the specific mutations, E55Q, Y125F, Q^122^FM(Y)F^126^. RDCs were recorded on ^15^N-labeled eRF1 N-domain weakly aligned in either 5% w/v *n*-octyl-penta (ethylene glycol)/octanol (C8E5) or the filamentous phage *Pf1*. These data indicate that the mutations alter the conformation and dynamics of the GTS loop that is distant from the mutation sites. We propose that the GTS loop forms a switch that is key for the multiple codon recognition capability of eRF1.

Eukaryotic protein synthesis is comprised of three stages namely, initiation, elongation and termination which results in the release of the nascent peptide. The termination of protein synthesis occurs when one of three stop codons (UAA, UAG or UGA) enters the ribosomal A site where it is decoded by a class I release factor (e.g. eRF1). Subsequent to recognition, peptidyltransferase RNA is hydrolyzed at the ribosome, a step mediated by the class II release factor (eRF3)^1^,^2^. The efficiency of termination is dependent on the formation of a quaternary complex that involves the ribosome, both the release factors (eRF1 and eRF3) and GTP^3^,^4^. Unlike prokaryotes, eukaryotes use a single omnipotent release factor, eRF1 capable of recognizing all three stop codons at the A site^5^,^6^. Despite extensive work, the principal mechanism of stop codon recognition in eukaryotes has still not been established. Chavatte *et al.* found via crosslinking experiments that eRF1 dictates specificity for stop codon recognition, a finding supported in part by mutagenesis analyses^7^. Together, these experimental observations established the region of eRF1 responsible for recognition of stop codons^8^,^9^. The crystal structure of human eRF1 revealed that the protein is comprised of an N-terminal (N), middle (M), and C-terminal (C) domains, all of which have been studied extensively over the past several years^10^. The C-domain constitutes the region that binds to eRF3^5^,^11^,^12^,^13^,^14^,^15^. The M-domain mimics the tRNA acceptor stem with a highly conserved GGQ motif, and is essential for ribosome binding and nascent peptide release via peptidyltransferase activity^10^,^16^,^17^. Based upon mutagenesis and sequence analysis, the N-domain is believed to be the site of codon decoding^7^,^8^,^18^. This domain contains the highly conserved NIKS motif (residues 61–64), YxCxxxF motif (residues 125–131)^19^ and the GTS loop (residues 31–33), which have all been implicated in stop codon recognition^9^,^15^,^20^,^21^. These elements are widely distributed throughout the protein structure confounding the identification of their precise roles in the stop codon recognition.

Highly conserved residues such as E55 and Y125 (Fig. 1), which are conserved in both eukaryotic and archeal release factors, have been implicated in models for eRF1 decoding site recognition. Further, the location of these residues on the surface of the N-domain may represent possible sites of interactions with mRNA^20^. Both E55 and Y125 have been shown to participate in the recognition of stop codon based on indirect^8^,^18^ and direct evidence^20^. Point mutations introduced at these positions revealed that Y125 is essential in maintaining the structure of the decoding site *via* its interactions with E55, and both residues are required for recognition of guanidine from the UAG codon. It is thought that the hydrogen bonds formed between these two residues are essential for the UAG-dependent release-factor activity. The effects of these mutations on the activity of release factors are summarized in Table 1 and shown on the structure of eRF1 in Fig. 1.

Chimeric proteins containing the *Stylonychia* eRF1 N-domain and the M- and C-domains of human eRF1 are able to specifically recognize only the UGA stop codon, further evidence that the N-domain is the site of stop codon recognition^22^. Additionally, chimeras with swapped ciliate and human N-domains^19^ highlight the importance of the QFM tripeptide in this UGA specificity, whereas chimeras of *Paramecium* eRF1 showed UGA specificity to be confined to the NIKS (residues 61–64) and YxCxxxF (residues 125–131) motifs^22^. The relevance of the GTS loop in stop codon recognition has also been addressed. Liang *et al.*^21^, hypothesized that five conserved amino acid sites (G31, T32, I62, K63, and C127) and three class-specific sites (G57, S70, and L126) trigger stop codon recognition. The codon specificity of mutations at T32, I35, E55, V71, and C127 imply that they are crucial for stop codon recognition^21^. The crystal structure of the eRF1 and eRF3 complex indicates that these residues, based on their polarity, may define the mRNA recognition pocket^15^.

We have previously analyzed Q^122^FM(Y)F^126^ and the wild-type eRF1 N-domains by solution state NMR^23^. Here we present a more complete comparison that includes residual dipolar coupling (RDC) data for all the mutants and wild-type elucidating the structural response induced by these mutations. The results show that although the global structure of the N-domain is conserved in all mutants, specific conformational perturbations are observed in the GTS loop, which is remote from the mutation sites. These observations further indicate that switching between omnipotency and unipotency in eRF1 may be modulated by distinct conformations of the GTS loop, which in turn are determined by the global structure of the N-domain and perhaps might be altered by the interactions with other components of the translation termination machinery.

## Results

### CD spectroscopy of eRF1 mutants

**Figure 2:**
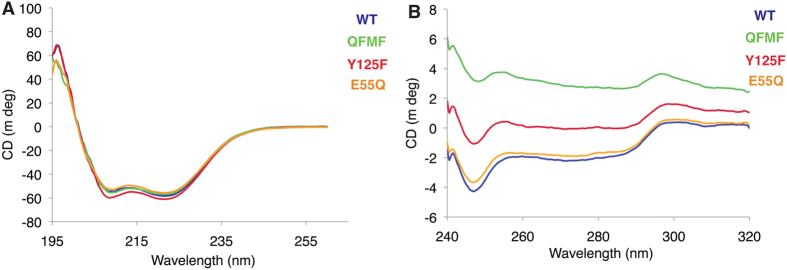
CD spectroscopy of wild-type eRF1 and its mutants, E55Q, Y125F and Q^122^FM(Y)F^126^ were measured at 25 °C on a Chirascan spectrophotometer (Applied Biophysics) in (**A**) far UV region and (**b**) near UV region depicting changes in the tertiary fold as a result of mutations. All samples were analyzed in NMR buffer (20 mM MES-K, pH 6.0, 100 mM KCl).

The secondary structure of the wild-type and mutant (E55Q, Y125F and Q^122^FM(Y)F^126^) eRF1 N-domains was measured using circular dichroism (CD) spectroscopy. The samples were dissolved in the same buffer as used for NMR spectroscopy (20 mM MES-K pH 6.0, 100 mM KCl). Figure 2A illustrates the overlaid far-UV CD spectra of the wild-type and mutant eRF1 N-domain constructs, measured at room temperature. The amplitudes of the CD spectra at the minima wavelengths 208 nm and 222 nm, and the maxima at 197 nm are indicative of similar α-helical content in the proteins. Overall, the CD measurements indicate that introduction of mutations to the protein has not significantly distorted or changed the secondary structure relative to the wild-type. In contrast, the near-UV CD spectra (Fig. 2B) may indicate alterations in the local environment of the aromatic amino acids suggesting differences in tertiary structure between the wild-type and mutants. The aromatic amino acid side chains are known to absorb in the 250–290 nm range^24^ and these absorption signals can be used as characteristic fingerprints of local protein structure after correction for the number of aromatic amino acids^24^ present in different mutants. The alterations are most significant for Q^122^FM(Y)F^126^, where the changes observed in the tertiary structure are a concerted result of several mutations propagating away from the mutation cluster. Although the CD spectra from each mutant are similar to the wild-type, a difference in ellipticity is observed, primarily due to the increased number of phenylalanine residues in Q^122^FM(Y)F^126^ and Y125F. The fact that these mutants have retained RF activity (to some extent) indicates their structural similarities, and the similar CD profiles support this observation.

### Structure determination of E55Q and Y125F

**Figure 3:**
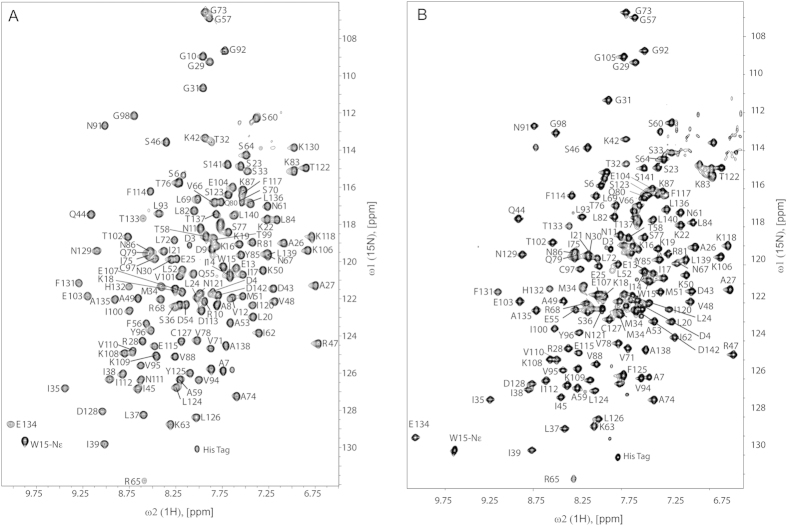
2D [^15^N,^1^H]-TROSY at 700 MHz proton frequency of mutant eRF1 (**A**) E55Q and (**B**) Y125F in 20 mM MES-K (pH 6.0), 100 mM KCl, 90% H_2_O/10% D_2_O at 298 K. Residue specific assignments of the amide resonances are labeled.

**Figure 4:**
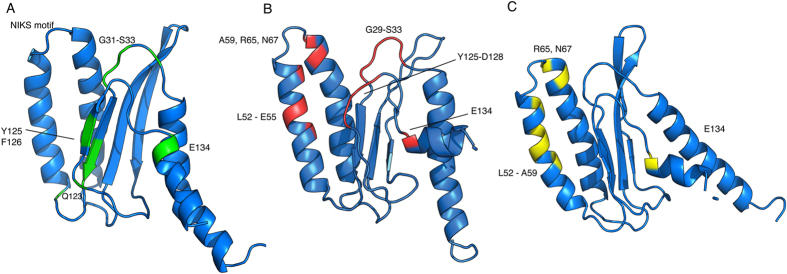
Structure of N-domain eRF1 mutants, (**A**) Q^122^FM(Y)F^126^ (structure derived from PDB: 2LGT) (**B**) Y125F (PDB: 2MQ6) and (**V**) E55Q (PDB:2MQ9) with the regions most affected by mutations highlighted.

The 2D [^15^N,^1^H]-TROSY spectra of E55Q and Y125F (Fig. 3) showed good spectral dispersion indicating the constructs were well folded which enabled the near-complete assignment of the ^1^H,^15^N and ^13^C resonances. The spectra exhibit similar peak patterns relative to the wild-type suggesting that the overall fold of the mutants is similar to the wild-type. To further investigate the structural changes inflicted by these mutations, we solved the solution structures of all the mutants. Based on the backbone assignment, the secondary structure was predicted and was found to correlate well with the wild-type, consistent with the CD data. The structures were calculated using unambiguous intramolecular NOEs, and hydrogen bonds extracted from the crystal structure as restraints, and subsequently refined against the experimental RDCs (Fig. 4). The backbone and heavy atom pairwise RMSD are 0.69 and 1.20 Å for E55Q, 0.81 and 1.22 Å for Y125F (Table 2), and as previously reported 0.26 and 0.68 Å for Q^122^FM(Y)F^126 23^. The coordinates and NMR constraints have been deposited in the protein data bank as 2MQ9 (E55Q) and 2MQ6 (Y125F). A comparison of the resulting structures shows that the beta-strands in both E55Q and Y125F are more structurally variable in comparison to the wild-type and Q^122^FM(Y)F^126^ due to fewer and weaker NOEs found in the regions surrounding the mutation sites. This might be attributed to a destabilization of the protein structure due to the mutations^20^,^23^. Table 3 lists the local RMSD values^25^ of Cα, backbone, heavy atoms and all atoms. The RMSD values for all mutants are within 1.0 Å, suggesting global preservation of the overall structure across all protein variants.

### Chemical shift perturbations (CSP) linked to mutations

**Figure 5:**
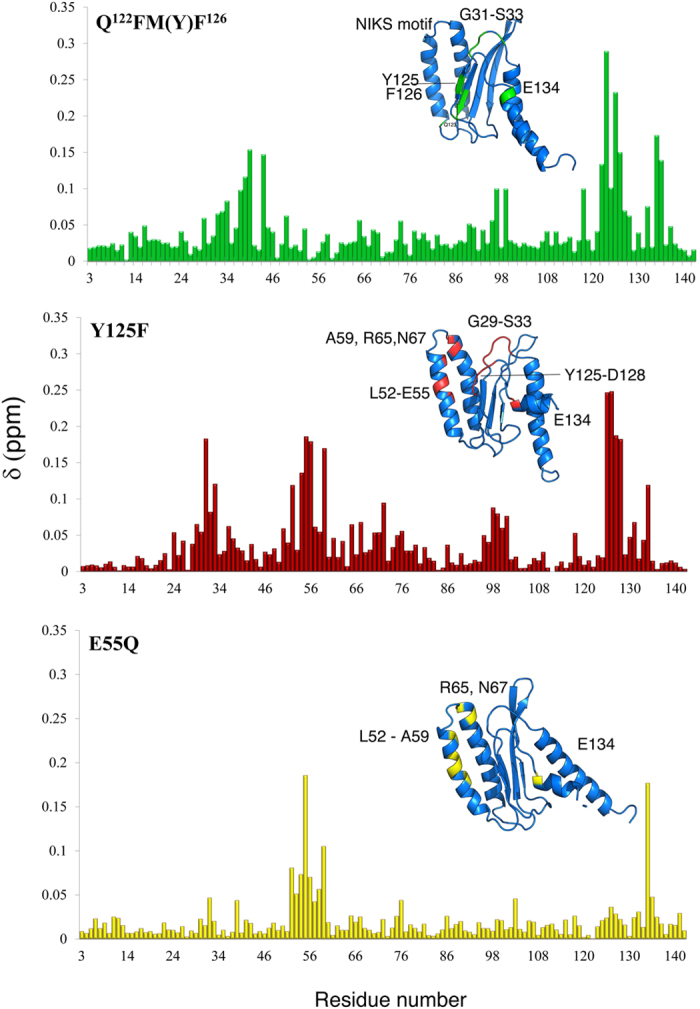
Chemical shift perturbation induced to the backbone amide signals between wild-type and mutant eRF1 N-domains, (**A**) Q^122^FM(Y)F^126^ (**B**) Y125F and (**C**) E55Q. Location of residues that experience significant perturbations upon introduction of mutation are highlighted on the structure (insert).

The overall CSPs observed between the wild-type and mutants are minor, with notable perturbations observed at and near the mutations, as expected. Interestingly, CSPs are also observed for residues that are sequentially distant from the mutations. Specifically, these regions are the highly conserved NIKS and YxCxxxF motif, which have been reported to influence stop codon recognition at the small ribosomal subunit^26^,^27^. Introduction of the Q^122^FM(Y)F^126^ mutations into human eRF1 converts its function from omnipotent to unipotent, thus leading to the hypotheses that the residues from positions 122–132 are not only essential for purine discrimination but also stop codon recognition^19^,^20^,^22^. Based on this observation, we investigated whether a correspondence exists between wild-type eRF1 and the mutant Q^122^FM(Y)F^126^ with respect to their backbone chemical shifts^23^. A general trend in the CSP profile is observed for Q^122^FM(Y)F^126^ compared to wild-type eRF1 (Fig. 5). The resonances stemming from the residues Q122, Y125 and F126 are observed to experience significant perturbations (0.15 < CSP < 0.3 ppm), as expected. Interestingly, these mutations also influenced other regions of the protein resulting in large CSPs in residues G29, G31-S33 (GTS loop), L37-I39, and Q44, indicating significant changes in the magnetic environment of these residues. The C-terminal tail of Q^122^FM(Y)F^126^ experienced deviations at E134 and A135 resulting in shifts of more than 0.1 ppm.

Both E55 and Y125 are associated to the recognition of G in UGA, with Y125 playing the dominant role of recognition. The hydrogen bond formed between the side chains of these two residues may help in maintaining the spatial proximity of the protein and thus influence RF activity^20^ as well as being part of the YxCxxxF motif. Comparison of the CSP profiles of Y125 and Q^122^FM(Y)F^126^ show that residues at the YxCxxxF motif (residues 125–131) may play a role in stop codon recognition via the GTS loop. In addition to the similar CSPs at the GTS loop (positions 29–33), larger perturbations in Y125F construct are observed at L52, D54, E55, A59, R65, and N67, residues forming the NIKS tetrapeptide motif. Interestingly, in the region of the mutation, Y125F, CSPs of Y125-D128 (the YxCxxxF motif) and E134 exhibit large variations, leaving the resonances from residues T122-L124 less perturbed. Likewise, the CSP profile of E55Q is similar to Y125F but not Q^122^FM(Y)F^126^, with only small perturbations observed in the GTS loop (0.05 ppm) (Fig. 5). However, all three mutants are perturbed at E134 regardless of the site and number of mutations.

The alternating conformations of the GTS loop might be regulated via the network of hydrogen bonds at the β4 hydrophobic core of the N-domain. Mutations in this region may disrupt this network and perturb the local structure^23^. In Q^122^FM(Y)F^126^ the L126F mutation induces flipping of the phenylalanine aromatic ring in the opposite direction as observed in the wild type, repositioning α-helix 3 closer to the GTS loop^23^. The Y125F mutation results in somewhat similar CSPs as observed in Q^122^FM(Y)F^126^, supporting the fact that the hydrogen network in this region might be perturbed in a similar fashion (Fig. 4)^23^.

### Global structural perturbations introduced by mutations

**Figure 6:**
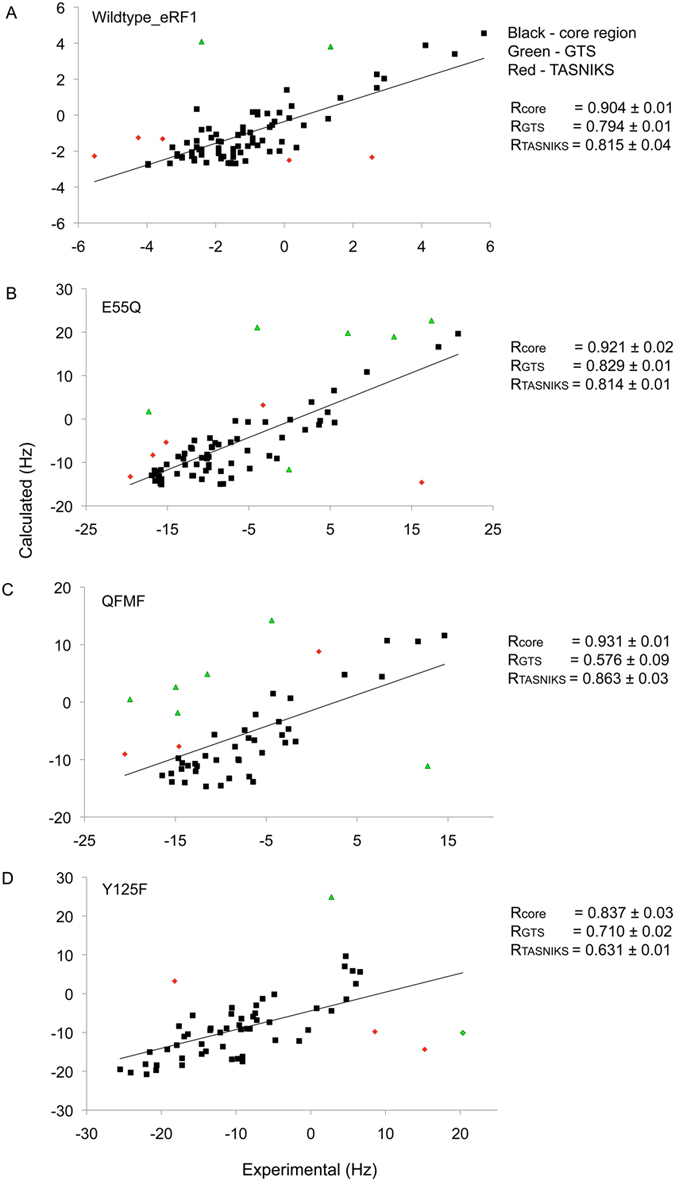
RDC experiments of wild-type and mutant eRF1 in C8E5 alignment media. Experimental and calculated residual dipolar coupling (RDC) for wild-type eRF1 and their correlation factors with respect to the full-length (**A**) wild-type eRF1 crystal structure without the GTS and NIKS regions, core (black), with the GTS loop included (green) and with the NIKS region included (red) using PALES. The same as observed for (**B**) E55Q and (**C**) Q^122^FM(Y)F^126^ (**D**) Y125F. The correlation coefficient for each protein is listed as R_CORE_, R_GTS_ or R_NIKS_.

**Figure 7:**
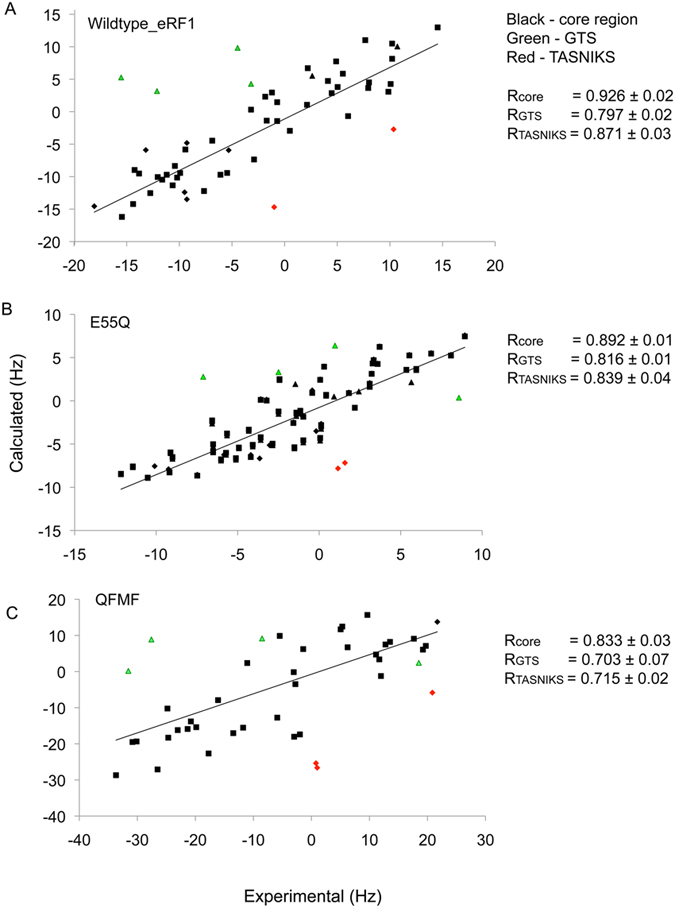
RDC experiments of wild-type and mutant eRF1 in *Pf1* alignment media. Experimental and calculated residual dipolar coupling (RDC) for wild-type eRF1 and their correlation factors with respect to the full-length (**A**) wild-type eRF1 crystal structure without the GTS and NIKS regions (black), with the GTS loop included (green) and with the NIKS region included (red) using PALES. The same as observed for (**B**) E55Q and (**C**) Q^122^FM(Y)F^126^. The correlation coefficient for each protein is listed as R_CORE_, R_GTS_ or R_NIKS_.

The comparison of the global structural response of the N-domain to the mutations may potentially indicate repositioning of many critical residues in the protein surface involved in the stop codon recognition. The dynamic properties of the wild-type and Q^122^FM(Y)F^126^ do not differ significantly from each other, implying that although the switching between omnipotency and unipotency of eRF1 can be explained by changes in the GTS-loop conformation, it is not reflected by the fast dynamics (ps-ns timescale)^23^. The absence of changes in the protein dynamics enabled us to employ backbone ^1^H-^15^N RDCs to assess global structural perturbations introduced by mutations^28^. The similarity of the experimental RDCs in the wild-type and all mutants indicate that the global structure is maintained, as observed in all 4 proteins (Fig. 4). However, the values of RDCs in the mutants deviate from RDCs back-calculated from the wild-type structure at the GTS and NIKS regions. These RDCs were analyzed using the crystal structure of wild-type eRF1 (PDB: 1DT9) and solution structure of the mutants Q^122^FM(Y)F^126^ (PDB: 2LGT), E55Q (PDB: 2MQ9) and Y125F (PDB: 2MQ6) as illustrated in Figs 6 and 7. The initial low correlation coefficients between experimental and structure-based calculated RDC values led us to remove RDCs corresponding to the GTS loop and the NIKS regions significantly improving the correlation. We then reanalyzed the R values using the same alignment tensor but with the inclusion of the GTS loop and NIKS region-derived RDCs to observe the deviation of these specific RDCs from those previously calculated.

For the wild-type N-domain in the C8E5 alignment medium, the correlation coefficient of R = 0.904 was observed for RDCs without the GTS loop and NIKS region. R = 0.794 and 0.815 were observed when GTS loop and NIKS region were included, respectively (Fig. 6). A similar pattern was observed for the wild-type N-domain in the *Pf1* alignment medium, with the correlation coefficient of R = 0.926 (core), and R = 0.797, R = 0.871 when the GTS loop and NIKS region were included, respectively (Fig. 7). Based on two different alignment media, e.g. C8E5 (Fig. 6) and *Pf1* (Fig. 7), we observed a notable correlation between the RDC data sets for all mutants with the slightly lower overall correlation coefficients for *Pf1*. The higher degree of alignment for all the eRF1 mutants (specifically Y125F) in the *Pf1* medium led to additional ^1^H-^1^H RDCs, which, in turn, resulted in the observed line broadening for many of the ^1^H-^15^N resonances. This broadening of NMR signals leads to a reduction in the resolution and sensitivity of the signals and larger experimental errors^29^,^30^. The increased noise in the ^1^D_HN_ dataset in the *Pf1* alignment medium resulted in an overall decrease in the correlation coefficient relative to C8E5. However, for the Q^122^FM(Y)F^126^ mutant, a lower value of calculated correlation coefficient (GTS loop omitted) was noticed in both employed alignment media. We have included the standard error of the mean correlation coefficient values estimated from the spectral noise-based variation of RDC coupling constants in the experimental dataset^31^. Based on the observed standard error, the R values reported for Q^122^FM(Y)F^126^ mutant fall within the range of the standard error. We attributed the observed difference in the correlation coefficients to the degree of the alignment induced by C8E5 relative to *Pf1*. We surmise that the higher degree of alignment by *Pf1* may lead to a stronger effect towards the GTS loop in the mutant thus causing the observed decrease in the correlation coefficient calculated for Q^122^FM(Y)F^126^.

For mutants E55Q and Y125F, the NIKS region seemed to be more affected and giving the lower correlation coefficient compared to the GTS loop. Figures 6 and 7 illustrate the difference between observed and experimental RDC values induced by the mutations to the GTS loop and NIKS regions of the N-domain in both alignment media. The CSP profiles of E55Q and Y125F show that most of the chemical shifts perturbations are located in these particular regions. A reduced correlation coefficient for the full structure relative to the selected regions further hints at the dynamic characteristic of both these regions. Thus, the lower correlation coefficients of RDC together with CSP data might indicate underlying local conformational variation in the protein in the dynamic range faster than the ^1^H chemical shift time scale.

## Discussion

In an earlier report we demonstrated that the selectivity of stop codon recognition might be governed by the multiple conformations adapted by the strictly conserved GTS loop^23^. This loop has been proposed to be involved either directly or indirectly in decoding and interacting with the stop codon of mRNA^27^,^32^. Further, from cross-linking studies^26^, the NIKS loop is assumed to interact with the first U of the stop codon via the anti-codon mimicry model proposed by Bertram *et al.*^10^,^33^. Similarly, mutational analysis and cross-linking studies have implicated the GTS motif in stop codon recognition^21^,^27^. From these studies came the proposal that although the NIKS loop is structurally remote from the GTS and YxCxxxF motifs, these fragments might still interact with stop codons within the ribosome. Although previous studies demonstrated that the GTS loop and the NIKS regions in the protein are involved in stop codon recognition, they have been studied separately and without atomic level details. Here we have extended the mutational analysis by demonstrating how these residues may structurally cross-talk leading to functionally relevant local and global changes in the conformation of the N-domain.

The CSP results show that a few mutations introduced onto the protein may cause significant structural changes at sites remote from the mutations, which, in turn, are related to the RF activity of the mutants. Mutational studies have been done on the residues constituting the hydrophobic core directly above the GTS loop, namely residues I35, V71, V78 and C127^8^,^15^ all of which affect the RF activity of the protein, clearly showing the importance of this loop. M51 and E55 on α -helix 2 are able to alter the stop codon recognition pattern^8^,^20^ and the NIKS motif of eRF1 was found to interact with eRF3 during decoding of UAA/UAG and UGA. Mutational analysis of these residues have shown that the release factor activity is affected when the hydrogen bond formation capability of the residues are altered. In fact, removing the donor proton capabilities of Y125 caused substantial reduction in the release activity, which reflects the importance of Y125 for preservation of the decoding site^20^. Although E55 and Y125 may be remote from each other in the sequence, the multiple structures of eRF1 N-domain show that when the protein is in its active conformation, these two residues are spatially close enough to form a hydrogen bond. It was found that UAG recognition was affected by substitutions at E55 and Y125 significantly diminishing the release factor activity. Similarly, the UAG release activity was found to be completely diminished^20^ in the Q^122^FM(Y)F^126^ mutant, and it has been shown that this region could be responsible for the recognition of the G base in the UAG stop codon. The CSP as well as the RDC profiles of both mutants, Y125F and Q^122^FM(Y)F^126^ are almost identical, especially in the responding regions remove from the points of mutation. Therefore, instead of alluding that only a single amino acid is responsible for the recognition of the G base, we conclude that it might be recognized by more than one amino acid in the YxCxxxF motif as well as more global conformational changes in the protein surface presented to mRNA. This is further supported by alterations in the RF activity observed in Q^122^FM(Y)F^126^ mutant largely insensitive to UAG and UAA stop codons. Seit-Nebi *et al.* reported mutational analysis of the YxCxxxF region that suggested the selectivity of the stop codon recognition, namely the A base in the second position is dependent on the residues present in this region as well as NIKS motif^9^,^19^. Our results support these conclusions.

In summary, despite a number of studies attempting to correlate individual residues in eRF1 with the release factor activity, a comprehensive structural model capable of explaining the accumulated mutational data is largely absent. Here we report NMR structures of several mutant forms of the N-domain of eRF1 exhibiting different specificities towards stop codons, which might serve as a basis for constructing such a model. Employing the RDC data from the two different sets of alignment media (C8E5 and *Pf1*) we refined our NMR structures and exposed the dynamic nature of the GTS loop^23^ that plays a key role in stop codon recognition. However, we are unable to conclude specifically that these variations occur at the μs-ms timescale based on our collected data. We showed that although these mutations are remote from the GTS loop and NIKS motif, they are able to structurally influence them, suggesting these regions to be highly dynamic in nature and as previously shown, involved in the recognition of stop codons^3^,^13^ (supplementary information). All these observed interactions point towards a higher degree of complexity in the stop codon decoding mechanism of eRF1 requiring interactions between different regions to modulate conformational changes, which may serve as a prerequisite for the translation termination to occur.

## Methods

### Expression and purification of protein samples

DNA encoding wild-type and mutants (Q^122^FM_F^126^, Y125F and E55Q) of the eRF1 N-domain were cloned into pET23( + ) expression vector (Novagen) with a hexa-histidine tag. Plasmids were transformed into competent *E.coli* BL21(DE3) cells and the resultant expression strain was grown in M9 minimal medium containing 1 mM of both ampicillin and chloramphenicol, supplemented with ^15^NH_4_Cl (1.0 g/l) and ^13^C_6_-glucose (2.0 g/l) as the sole nitrogen and carbon sources (Cambridge Isotope Laboratories). Cultures were incubated at 37 °C, shaking at 180 rpm to an A_600_ of 0.8. The culture was cooled to room temperature before adding 1 mM isopropyl 1-thio-β-D-galactopyranoside, and incubated overnight at 20 °C. The cells were harvested by centrifugation and resuspended in 20 mM sodium phosphate, pH 6.5, 100 mM KCl. Bacterial lysis was performed by sonication for 30 minutes of 2 sec burst/3 sec rest. The resulting suspension was again centrifuged and the cell lysate was purified via affinity chromatography using a 5 ml HisTrap HP column (GE Healthcare). Bound protein was eluted using a 20 mM sodium phosphate, pH 6.5, 100 mM KCl containing 500 mM imidazole. The appropriate fractions were pooled and imidazole removed using three 5 ml HiTrap desalting columns (GE Healthcare) connected in series, into 20 mM MES pH 6.0, 100 mM KCl, 2 mM DTT (NMR buffer). The fractions containing eRF1 were pooled and concentrated to approximately 1 mM using Centricon YM3 devices (molecular weight cutoff 10 000) (Amicon). Protein yield was calculated by measuring the absorbance at 280 nm on NanoDrop™ spectrophotometer (Thermo Scientific), with the corresponding molar extinction coefficient of each protein. The presence of eRF1 wild-type and its mutants (Q^122^FM_F^126^, Y125F, and E55Q) were detected throughout the protocol using 12% (w/v) polyacrylamide gel electrophoresis.

### Circular Dichroism Spectroscopy

All CD experiments were recorded at the pH of the corresponding NMR samples using a Chirascan CD spectrometer (Applied Photophysics, UK). Near and far-UV measurements (200–320 nm) were both performed using a quartz cell with a path length of 0.01 cm and the temperature was maintained at 25 °C and all spectra were corrected against the buffer signal. CD spectra were acquired using 70 μl of sample at concentrations of 100 μM in NMR buffer. Three replicates were acquired, averaged and corrected for the buffer blank and processing of spectra was done using the available software Chirascan.

### Alignment of eRF1-wt and mutants in Anisotropic Media

Alignment in the magnetic field was achieved at 20 °C using 5% w/v *n*-octyl-penta(ethylene glycol)/octanol (denoted as C8E5) (Sigma-Aldrich)^34^. Alternatively, at 25 °C, *Pf1* bacteriophage (10 mg/mL) (Hyglos GmbH) was used to achieve alignment^35^.

### NMR Measurements

All NMR spectra were acquired using 600 and 700 MHz Bruker Avance II spectrometers. A series of 2D [^15^N, ^1^H]-TROSY experiments were utilized to monitor the chemical shift perturbations (CSP) of the ^15^N, ^1^H spins. The chemical shifts were referenced directly (^1^H) relative to 4,4-dimethyl-4-silapentane-1-sulfonic acid (DSS). The NMR data were processed using TopSpin 2.0 (www.bruker-biospin.com) and analyzed using CARA (www.nmr.ch). The wild-type and mutants were dissolved in 20 mM MES pH 6.0, 100 mM KCl, 2 mM DTT (NMR buffer). The buffer components are kept consistent for all NMR experiments unless otherwise stated. The assignment process was facilitated by comparison with chemical shifts deposited in the Biological Magnetic Resonance Data Bank (www.bmrb.wisc.edu). Side chain ^1^H and ^13^C were assigned using iterative analysis of the 3D ^15^N-NOESY-HSQC and ^13^C-NOESY-HMQC spectra coupled with structure calculations. The weighted CSP for backbone ^15^N and ^1^H_N_ resonances were calculated by the equation^36^ Δδ = [(Δδ_HN_)^2^ + (0.1Δδ_N_)^2^]^0.5^. Residual dipolar couplings (RDCs) were derived from 2D [^15^N,^1^H]-TROSY and anti-TROSY experiments measured in both isotropic and anisotropic conditions, using uniformly ^15^N-labeled proteins in the same sample NMR buffer composition as mentioned above. The N-H RDCs were measured for the wild type and mutants by obtaining the differences in splitting between an aligned and an isotropic sample (the RDCs were not corrected for the negative gyromagnetic ratio of ^15^N). The axial and rhombicity of the alignment tensor were calculated using PALES^37^. The standard errors of the mean correlation coefficient values were estimated from the spectral noise-based dependent variation of RDC coupling constants in the experimental dataset.

### Structure Determination and Analysis

NOE distance restraints for all the calculated structures were obtained from ^15^N-NOESY-HSQC and ^13^C-NOESY-HMQC spectra, respectively. Backbone dihedral angle restraints (φ and ψ) were derived from backbone ^13^C’, ^13^C_α_, ^13^C_β_, ^1^H_α_ and ^1^H_β_ chemical shift values using TALOS^38^. Structure calculations were performed using CYANA 3.0^39^,^40^ and visualized using MOLMOL^41^ and PyMOL (Delano Scientific). The quality of the final structures was assessed using PROCHECK-NMR^42^.

## Additional Information

**Accession codes**: Y125F PDB ID code 2MQ6 and E55Q PDB ID code 2MQ9.

**How to cite this article**: Pillay, S. *et al.* Structural characterization of eRF1 mutants indicate a complex mechanism of stop codon recognition. *Sci. Rep.* **6**, 18644; doi: 10.1038/srep18644 (2016).

## Supplementary Material

Supplementary Information

## Figures and Tables

**Figure 1:**
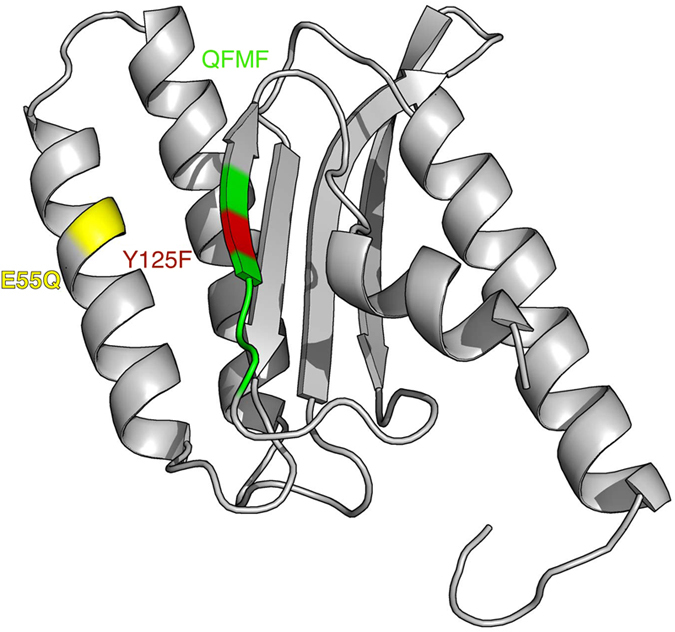
Activity associated mutations introduced in N-domain of eRF1. The E55Q mutation is colored yellow and believed to abolish hydrogen bonds with Y125F colored in red. The unipotent-associated mutation, Q^122^FM_F^126^ is colored in green. The coordinates for the N-domain are derived from crystallographic data^10^.

**Table 1:** *In vitro* release activity of N-domain eRF1 mutants (in percentage) to the wild-type eRF1.

Mutant/codon	UAA	UAG	UGA
F131A^a^	10	7	62
E55Q^b^	75	35	80
Y125F^b^	100	34	100
T112Q + S123F + L124M + L126F^a^	0	0	80

^a^The release activity of mutant eRF1s was measured according to the *in vitro* Caskey’s assay. Data referenced from^20^.

^b^Activity of mutants of N-domain in percent to the human wild-type eRF1 (Kisselev, private communication).

**Table 2:** Structure restraints and statistics for the selected 20 lowest energy structures of the eRF1 mutants E55Q and Y125F.

NMR restraints	E55Q	Y125F
Total unambiguous distance restraints	1892	1925
Intra residual	1371	792
sequential (|i-j| = 1)	294	580
Medium (2 < |i-j| < 5)	1338	266
Long range (|i–J| > 5)	528	276
Hydrogen bond restraints	106	106
Dihedral angle restraints	180	180
RDC restraints	30	40
Residual Dipolar Couplings R-factor (%)	0.953 +/− 0.005	0.994 +/− 0.001
Magnitude (Da)	10.6	−13.19
Rhombicity (R)	0.207	0.511
Structure Statistics		
NOE violations > 0.5 Å	1	1
Angle violations > 10 °	0	3
Max dipolar coupling violations (Hz)	0	3.17
RMSD from average atomic coordinates (residues 10–140, A)		
Backbone atoms	0.69 +/− 0.12	0.81 +/− 0.15
All heavy atoms	1.20 +/− 0.09	1.22 +/− 0.13
Ramachandran analysis (%)		
Residues in most favored regions	81.6	89.3
Residues in additional allowed regions	17.2	10
Residues in generously allowed regions	1.1	0.2
Residues in disallowed regions	0	0.5

**Table 3:** Pairwise backbone RMSD of mutants relative to the wild-type.

	Cα	Backbone	Heavy
Q^122^FM(Y)F^126^	0.744	0.746	1.211
E55Q	1.229	1.222	1.830
Y125F	0.869	0.86	1.273

## References

[b1] Nakamura Y., Ito K. & Isaksson L. A. Emerging understanding of translation termination. Cell 87, 147–150 (1996). 8861897 10.1016/s0092-8674(00)81331-8

[b2] Kisselev L., Ehrenberg M. & Frolova L. Termination of translation: interplay of mRNA, rRNAs and release factors? Embo Journal 22, 175–182 (2003). 12514123 10.1093/emboj/cdg017 PMC140092

[b3] Frolova L. *et al.* A highly conserved eukaryotic protein family possessing properties of polypeptide chain release factor. Nature 372, 701–703, 10.1038/372701a0 (1994). 7990965

[b4] Zhouravleva G. *et al.* Termination of Translation in Eukaryotes Is Governed by 2 Interacting Polypeptide-Chain Release Factors, Erf1 and Erf3. Embo Journal 14, 4065–4072 (1995). 7664746 10.1002/j.1460-2075.1995.tb00078.x PMC394485

[b5] Nakamura Y. & Ito K. How protein reads the stop codon and terminates translation. Genes to Cells 3, 265–278 (1998). 9685178 10.1046/j.1365-2443.1998.00191.x

[b6] Nakamura Y., Ito K. & Ehrenberg M. Mimicry grasps reality in translation termination. Cell 101, 349–352 (2000). 10830162 10.1016/s0092-8674(00)80845-4

[b7] Frolova L. Y., Merkulova T. I. & Kisselev L. L. Translation termination in eukaryotes: Polypeptide release factor eRF1 is composed of functionally and structurally distinct domains. Rna-a Publication of the Rna Society 6, 381–390 (2000). 10.1017/s135583820099143x PMC1369920 10744022

[b8] Bertram G., Bell H. A., Ritchie D. W., Fullerton G. & Stansfield I. Terminating eukaryote translation: Domain 1 of release factor eRF1 functions in stop codon recognition. Rna-a Publication of the Rna Society 6, 1236–1247 (2000). 10.1017/s1355838200000777 PMC1369997 10999601

[b9] Frolova L., Seit-Nebi A. & Kisselev L. Highly conserved NIKS tetrapeptide is functionally essential in eukaryotic translation termination factor eRF1. Rna-a Publication of the Rna Society 8, 129–136 (2002). 10.1017/s1355838202013262 PMC1370237 11911360

[b10] Song H. W. *et al.* The crystal structure of human eukaryotic release factor eRF1—Mechanism of stop codon recognition and peptidyl-tRNA hydrolysis. Cell 100, 311–321 (2000). 10676813 10.1016/s0092-8674(00)80667-4

[b11] Kononenko A. V. *et al.* Role of the individual domains of translation termination factor eRF1 in GTP binding to eRF3. Proteins-Structure Function and Bioinformatics 70, 388–393, 10.1002/Prot.21544 (2008). 17680691

[b12] Fan-Minogue H. *et al.* Distinct eRF3 requirements suggest alternate eRF1 conformations mediate peptide release during eukaryotic translation termination. Molecular Cell 30, 599–609, 10.1016/j.molcel.2008.03.020 (2008). 18538658 PMC2475577

[b13] Merkulova T. I., Frolova L. Y., Lazar M., Camonis J. & Kisselev L. L. C-terminal domains of human translation termination factors eRF1 and eRF3 mediate their *in vivo* interaction. Febs Letters 443, 41–47 (1999). 9928949 10.1016/s0014-5793(98)01669-x

[b14] Ebihara K. & Nakamura Y. C-terminal interaction of translational release factors eRF1 and eRF3 of fission yeast: G-domain uncoupled binding and the role of conserved amino acids. Rna-a Publication of the Rna Society 5, 739–750 (1999). 10.1017/s135583829998216x PMC1369801 10376874

[b15] Cheng Z. *et al.* Structural insights into eRF3 and stop codon recognition by eRF1. Gene Dev 23, 1106–1118, 10.1101/Gad.1770109 (2009). 19417105 PMC2682955

[b16] Frolova L. Y. *et al.* Mutations in the highly conserved GGQ motif of class 1 polypeptide release factors abolish ability of human eRF1 to trigger peptidyl-tRNA hydrolysis. Rna-a Publication of the Rna Society 5, 1014–1020 (1999). 10.1017/s135583829999043x PMC1369825 10445876

[b17] Seit-Nebi A., Frolova L., Justesen J. & Kisselev L. Class-1 translation termination factors: invariant GGQ minidomain is essential for release activity and ribosome binding but not for stop codon recognition. Nucleic Acids Res 29, 3982–3987 (2001). 11574680 10.1093/nar/29.19.3982 PMC60236

[b18] Inagaki Y., Blouin C., Doolittle W. F. & Roger A. J. Convergence and constraint in eukaryotic release factor 1 (eRF1) domain 1: the evolution of stop codon specificity. Nucleic Acids Res 30, 532–544 (2002). 11788716 10.1093/nar/30.2.532 PMC99827

[b19] Seit-Nebi A., Frolova L. & Kisselev L. Conversion of omnipotent translation termination factor eRF1 into ciliate-like UGA-only unipotent eRF1. Embo Rep 3, 881–886, 10.1093/embo-reports/kvf178 (2002). 12189178 PMC1084231

[b20] Kolosov P. *et al.* Invariant amino acids essential for decoding function of polypeptide release factor eRF1. Nucleic Acids Res 33, 6418–6425, 10.1093/Nar/Gki927 (2005). 16282590 PMC1283522

[b21] Liang H., Wong J. Y., Bao Q., Cavalcanti A. R. O. & Landweber L. F. Decoding the decoding region: Analysis of eukaryotic release factor (eRF1) stop codon-binding residues. J Mol Evol 60, 337–344, 10.1007/s00239-004-0211-8 (2005). 15871044

[b22] Lekomtsev S. *et al.* Different modes of stop codon restriction by the stylonychia and paramecium eRF1 translation termination factors. P Natl Acad Sci USA 104, 10824–10829 (2007). 10.1073/pnas.0703887104 PMC1904165 17573528

[b23] Wong L. E., Li Y., Pillay S., Frolova L. & Pervushin K. Selectivity of stop codon recognition in translation termination is modulated by multiple conformations of GTS loop in eRF1. Nucleic acids Res 40, 5751–5765, 10.1093/nar/gks192 (2012). 22383581 PMC3384315

[b24] Kelly S. P., NC. The use of Circular Dichroism in the investigation of protein structure and function. Curr Protein Pept Sci 1, 349–384 (2000). 12369905 10.2174/1389203003381315

[b25] Maiti R., Van Domselaar G. H., Zhang H. & Wishart D. S. SuperPose: a simple server for sophisticated structural superposition. Nucleic Acids Res 32, W590–594, 10.1093/nar/gkh477 (2004). 15215457 PMC441615

[b26] Chavatte L., Seit-Nebi A., Dubovaya V. & Favre A. The invariant uridine of stop codons contacts the conserved NIKSR loop of human eRF1 in the ribosome. The EMBO journal 21, 5302–5311 (2002). 12356746 10.1093/emboj/cdf484 PMC129024

[b27] Bulygin K. N. *et al.* Three distinct peptides from the N domain of translation termination factor eRF1 surround stop codon in the ribosome. Rna-a Publication of the Rna Society 16, 1902–1914, 10.1261/Rna.2066910 (2010). PMC2941099 20688868

[b28] Prestegard J. H., Al-Hashimi H. M. & Tolman J. R. NMR structures of biomolecules using field oriented media and residual dipolar couplings. Q Rev Biophys 33, 371–424 (2000). 11233409 10.1017/s0033583500003656

[b29] Ottiger M., Delaglio F. & Bax A. Measurement of J and dipolar couplings from simplified two-dimensional NMR spectra. J Magn Reson 131, 373–378 (1998). 9571116 10.1006/jmre.1998.1361

[b30] Tjandra N., Grzesiek S. & Bax A. Magnetic field dependence of nitrogen proton J-splittings in N-15 enriched human ubiquitin resulting from relaxation interference and residual dipolar coupling. J Am Chem Soc 118, 6264–6272 (1996).

[b31] Bax A., Kontaxis G. & Tjandra N. Dipolar couplings in macromolecular structure determination, Nuclear Magnetic Resonance of Biological Macromolecules Pt. B, 127–174 (2001). 10.1016/s0076-6879(01)39313-8 11462810

[b32] Bulygin K. N. *et al.* Adenine and guanine recognition of stop codon is mediated by different N domain conformations of translation termination factor eRF1. Nucleic Acids Res 39, 7134–7146, 10.1093/Nar/Gkr376 (2011). 21602268 PMC3167606

[b33] Ito K. *et al.* Omnipotent decoding potential resides in eukaryotic translation termination factor eRF1 of variant-code organisms and is modulated by the interactions of amino acid sequences within domain 1. P Natl Acad Sci USA 99, 8494–8499, 10.1073/pnas.142690099 (2002). PMC124286 12084909

[b34] Ruckert M. & Otting G. Alignment of biological macromolecules in novel nonionic liquid crystalline media for NMR experiments. J Am Chem Soc 122, 7793–7797, 10.1021/Ja001068h (2000).

[b35] Hansen M. R., Mueller L. & Pardi A. Tunable alignment of macromolecules by filamentous phage yields dipolar coupling interactions. Nat Struct Biol 5, 1065–1074 (1998). 9846877 10.1038/4176

[b36] Cavanagh J. Protein NMR spectroscopy : principles and practice. 2nd edn, (Academic Press, 2007).

[b37] Zweckstetter M. NMR: prediction of molecular alignment from structure using the PALES software. Nat Protoc 3, 679–690, 10.1038/nprot.2008.36 (2008). 18388951

[b38] Cornilescu G., Delaglio F. & Bax A. Protein backbone angle restraints from searching a database for chemical shift and sequence homology. J Biomol NMR 13, 289–302 (1999). 10212987 10.1023/a:1008392405740

[b39] Guntert P., Mumenthaler C. & Wuthrich K. Torsion angle dynamics for NMR structure calculation with the new program DYANA. J Mol Biol 273, 283–298 (1997). 9367762 10.1006/jmbi.1997.1284

[b40] Herrmann T., Guntert P. & Wuthrich K. Protein NMR structure determination with automated NOE assignment using the new software CANDID and the torsion angle dynamics algorithm DYANA. J Mol Biol 319, 209–227, 10.1016/S0022-2836(02)00241-3 (2002). 12051947

[b41] Koradi R., Billeter M. & Wuthrich K. MOLMOL: A program for display and analysis of macromolecular structures. Journal of Molecular Graphics 14, 51-& (1996). 8744573 10.1016/0263-7855(96)00009-4

[b42] Laskowski R. A., Rullmann J. A. C., MacArthur M. W., Kaptein R. & Thornton J. M. AQUA and PROCHECK-NMR: Programs for checking the quality of protein structures solved by NMR. J Biomol NMR 8, 477–486 (1996). 9008363 10.1007/BF00228148

