# Supplementary material for: Structural characterization of eRF1 mutants indicate a complex mechanism of stop codon recognition
Source: Sci Rep. 2016 Jan 4;6:18644. doi: 10.1038/srep18644 (PMC4698671; doi:10.1038/srep18644)
Supplement: Supplementary Information [file srep18644-s1.pdf]

# Structural characterization of eRF1 mutants indicate a complex mechanism of stop codon recognition

**Shubhadra Pillay [1,*], Yan Li[1], Leo E Wong[1], and Konstantin Pervushin[1,*]**

[1] School of Biological Sciences, Nanyang Technological University, 60 Nanyang Drive, Singapore 637551, Singapore

*Correspondence to kpervushin@ntu.edu.sg, shub0004@e.ntu.edu.sg

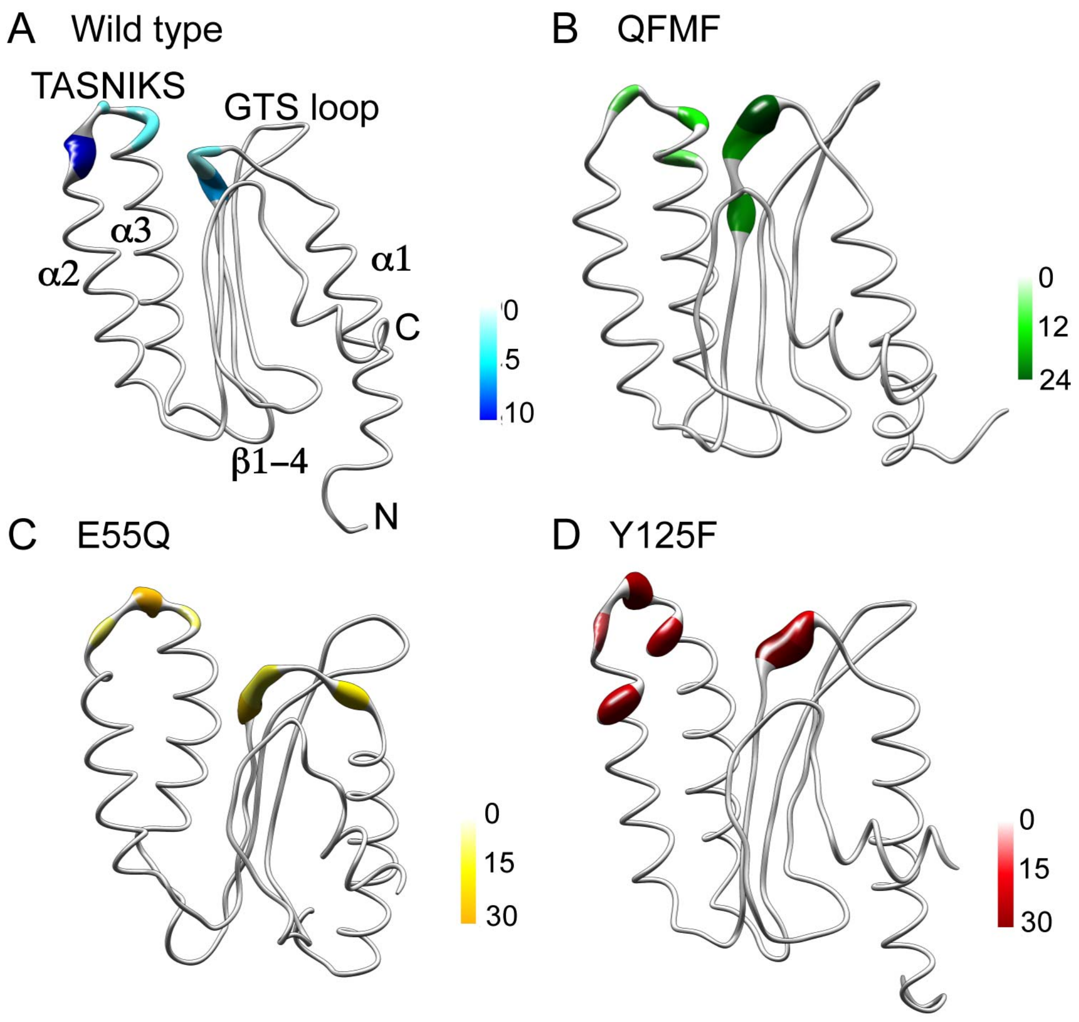


**Supplementary Info** Structural map of the difference between back-calculated and experimental RDC values (in Hz) induced by mutations on the GTS loop and TASNIKS region of N-domain eRF1 A) wt-eRF1, B) Q122FM(Y)F1126 C) E55Q and D) Y125F
